# Early- and intermediate-term results of surgical correction in 122 patients with total anomalous pulmonary venous connection and biventricular physiology

**DOI:** 10.1186/s13019-015-0387-6

**Published:** 2015-11-24

**Authors:** Keyan Zhao, Huishan Wang, Zengwei Wang, Hongyu Zhu, Minhua Fang, Xianyang Zhu, Nanbin Zhang, Hengchang Song

**Affiliations:** Department of Cardiac Surgery, Shenyang Northern Hospital, 83 Wenhua Road, Shenhe District, Shenyang, 110840 Liaoning Province China

**Keywords:** Congenital defects, Total anomalous pulmonary venous connection, Surgery, Regression analysis

## Abstract

**Background:**

We retrospectively reported our 26-year experience with operative repair of total anomalous pulmonary venous connection (TAPVC) with biventricular physiology.

**Methods:**

Between December 1982 and December 2008, 122 TAPVC patients with biventricular heart underwent surgical repair in our department. Moderate or deep hypothermia was induced at the time of cardiopulmonary bypass (CPB). Follow-up was conducted for 5 postoperative years. Surgical outcomes of early and intermediate deaths after TAPVC repair were retrospectively analyzed.

**Results:**

Six deaths occurred operatively; and three deaths, during follow-up. The 5-year survival rates after TAPVC repair was 92.6 %, without gradient across the anastomosis. The survival rate of the patients who were younger was 78.8 %, significantly lower than those older than 1 year. It was also lower in those who were less than 6 kg in weight. Three patients died during follow-up. Three patients died of ventricular arrhythmia, right heart failure, and pneumonia, respectively, during follow-up. If the left atrium pressure was higher than 15 mm Hg, the snare of the vertical vein was loosened after CPB ceased in the patients with supracardiac connection. It decreased from 21 ± 5 to 13 ± 3 mm Hg. The vertical vein was ligated in 57 cases and left open in 20 cases. A patient with an intact vertical vein had a large shunt and was cured by intervention afterward. Supraventricular arrhythmia occurred in 19 patients with the supercardiac type repaired through a biatrial incision. One patient died of ventricular arrhythmia, and none of the remaining patients had arrhythmias.

**Conclusion:**

Surgical treatment of TAPVC carried a low operative risk and had satisfactory immediate and intermediate results. Age younger than 1 year and weight less than 6 kg were risk factors. It was a good choice to leave the vertical vein open in the patients with a left atrial pressure higher than 15 mm Hg.

## Background

Total anomalous pulmonary venous connection (TAPVC) is a relatively uncommon anomaly in which connection between the pulmonary veins and the left atrium is absent, accounting for only about 1.5 % to 3 % of cases of congenital heart disease [1, 2]. The pulmonary veins establish an anomalous connection that delivers pulmonary venous blood to the right side of the heart rather than to the left side. A patent foramen ovale or an atrial septal defect provides a right-to-left shunt so that an admixture of oxygenated and unoxygenated blood eventually reaches the left side of the heart. Without the right-to-left shunting of blood, an infant would die [3]. It is a life-threatening congenital cardiac malformation that requires surgical therapy. It can be categorized according to the site of drainage into the systemic circulation (supracardiac, 45 %; infracardiac, 25 %; cardiac, 25 %; mixed, 5 %) [4]. Surgical repair is best performed at the time of diagnosis, as an elective procedure, to avoid potential long-term problems associated with right heart volume and pressure overload, or long-standing cyanosis. The surgical correction of TAPVC has become increasingly successful owing to early referral, improved surgical and anesthetic techniques, and improved intraoperative and postoperative care.

While surgical repair of TAPVC has historically been associated with a significant risk of early mortality, with reports in the literature ranging from 10 % to 50 % [5, 6]. Many reports have argued that among patients with congenital heart defects, those with TAPVC and single-ventricle physiology have the poorest survival [7–9]. These patients undergo procedures including Fontan, bidirectional Glenn, systemic-pulmonary shunt, or pulmonary artery banding, obviously with high operative mortality and poor long-term survival [4, 10]. Patients with biventricular physiology had good results, but 1-month survival was 90 % in a previous study [5] and 95.1 % at our center. Earlier studies indicated that biventricular patients with preoperative pulmonary venous obstruction were at a high risk of post-repair pulmonary venous obstruction, which this has been neutralized as a risk factor in some more-recent studies [11]. We have performed operations for TAPVC in our center since 1982. Patients with single-ventricle physiology were fewer because of reasons such as economic status and age. We argued it in single ventricular operation. We obtained better results in patients with biventricular physiology. However, whether the vertical vein should be ligated at the time of total anomalous pulmonary venous repair is controversial. Herein, we report retrospectively our 26-year experience with TAPVC and biventricular physiology, including clinical presentations, operative techniques, results, and survival. In addition, we discussed how to manage the vertical vein.

## Methods

### Patients

Between December 1982 and December 2008, 122 patients with TAPVC and biventricular hearts underwent surgical repair at Northern Hospital, Liaoning province, China. We retrospectively evaluated these patients, whose characteristics (54 males and 68 females) are summarized in Table 1. The mean age of the patients at operation was 8.5 ± 7.0 years (27 days to 44 years), with 18 patients younger than 1 year. The mean body weight was 20.1 ± 14.6 kg (from 2.7 to 87 kg), with 12 patients less than 6 kg in weight. Of the patients, 112 (91.8 %) had symptoms of tachypnea, cyanosis, or failure to thrive. They were diagnosed by using echocardiography, cardiac catheterization and cineangiography, or computer tomography. TAPVC was classified as supracardiac in 77 patients (63.1 %), cardiac in 39 patients (32.0 %), infracardiac in one patient, and mixed in 5 patients (4.1 %). Twenty-one patients required preoperative inotropic support, and 10 required mechanical ventilation. Emergency surgery was performed in 8 patients, of whom 7 were younger than 1 year and 6 were weighed less than 6 kg. All of the patients who underwent emergency surgery had severe dyspnea and continuous metabolic acidosis before the operation. Associated malformations included patent ductus in 21 patients, pulmonary stenosis in 5 patients, and moderate or severe tricuspid regurgitation in 28 patients. Twenty-five patients had moderate pulmonary artery hypertension (PAH) and 27 had severe PAH.

**Table 1:** Patient characteristics

Variable	No. or mean ± SD
Patients, total	122
Sex	
Male	54
Female	68
Age at operation, year	8.5 ± 7.0
Age ≤ 1 year	18
Body weight	
At operation, kg	20.1 ± 14.6
<6 kg	12
Type	
Supracardiac	77
Cardiac	39
Infracardiac	1
Mixed	5
Associated malformations	
Patent ductus	21
Pulmonary stenosis	5
Moderate PAH	25
Severe PAH	27

### Surgical technique

All of the operations were performed under general anesthesia through median sternotomy. Cardiopulmonary bypass (CPB) was commenced with ascending aortic perfusion and bicaval cannulations. Moderate or deep hypothermia was induced. Measures to ensure myocardial protection included cold blood cardioplegia infusion.

In the patients with supracardiac connection, biatrial incision (*n* = 66) or the modified superior approach [12] were used. In patients with supracardiac or infracardiac connections, the snare of the vertical vein was loosened if the left atrium pressure was higher than 15 mm Hg after CPB ceased in the patients with supracardiac connection. Left atrial pressures (LAPs) were recorded before and after the snare was loosened. The vertical vein was ligated in 57 patients and left open in 20 patients. In the patient with infracardiac connection (*n* = 1), the apex of the heart was tilted up to the right and then the vertical vein was ligated and incised to the diaphragm. The common pulmonary venous chamber was anastomosed to the back of the left atrium. Meanwhile, the associated malformations were handled at the operation.

The patients were followed up in the outpatient department by clinical examination, echocardiography, and electrocardiography for 5 years after repair.

### Statistical analysis

Quantitative data were expressed as mean ± the standard deviation values as appropriate. The end point of follow-up was TAPVC repair until the date of death, lost to follow-up, or 5 years after the operation. All *P* values were two-sided, and a *P* < 0.05 was considered statistically significant. Age (younger than 1 year), sex, and type (supracardiac or infracardiac, for cardiac and mixed cases were few) were determined by using the chi-square test. Quantitative data (weight, bypass time, and clamp time) were analyzed by using a *t* test. LAPs before and after the snare was loosened were analyzed by performing a paired *t* test. Statistical analysis was performed with the SPSS Version 13.0 software package (SPSS Inc, Chicago, IL).

## Results

Six early deaths (4.9 %) occurred. One patient with supracardiac connection died intraoperatively from failure to successfully wean from the CPB. The patient was a 3-month-old infant. Owing to severe dyspnea and continuous metabolic acidosis, mechanical ventilation was applied preoperatively. Emergency surgery was performed. During the time of bypass assistance, the heart contracted weakly and oxygenation worsened. Despite various attempts, the patient could not be weaned from CPB. The reasons may be the poor preoperative status, systemic inflammatory response syndrome, or poor cardiac protection. Two patients with severe pulmonary artery hypertension, whose pulmonary vascular resistance values were 20 and 13.4 Wood units, respectively, died from low cardiac output. One died of ventricular fibrillation 15 days after the repair, and the other two died of pulmonary infection and respiratory failure 30 days after the repair. One month after the repair, 116 patients survived.

**Fig. 1:**
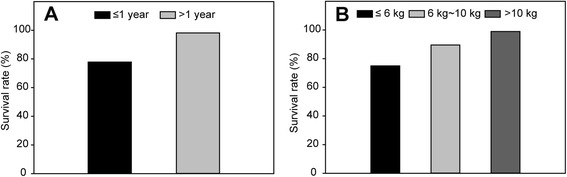
Survival rate of patients with different age and body weight. **a** Survival rate of patients younger than one year is much lower than those older; **b** Survival rate was 77.8 % in patients who were ≤6 kg in weight. It was much lower than that of patients who were 6 ~ 10 kg or >10 kg in weight

Among the 18 patients younger than 1 year, four died. However, among the remaining 104 patients, only two died later. The results of the statistical analysis showed that the patients younger than 1 year had a higher mortality risk than the others (*P* = 0.000; Fig. 1a). No statistically significant differences were observed among the patients in terms of sex and type (supracardiac or cardiac).

The survival rate was 75 % in the patients who weighed less than 6 kg. In the *t* test, weighing less than 6 kg was also a risk factor (*P* = 0.001). The risk was 89.5 % in those weighing 6–10 kg (Fig. 1b). Among the 27 patients with severe PAH, 3 died. Their mortality was significantly higher than that of the patients with moderate PAH (1/25 patients). The mean duration of CPB was 95.3 ± 33.9 min (range, 35.0–227.0 min), and the mean aortic cross-clamp time was 52.5 ± 22.2 min (range, 12–123 min). Hospital death was not correlated with bypass time (*P* = 0.441) or cross-clamp time (*P* = 0.772).

Emergency surgery was performed in 8 patients, of whom 7 were younger than 1 year and 6 weighed less than 6 kg. This is the reason why the mortality rate was 22.2 % in the younger patients and 25 % in the patients who weighed less than 6 kg.

**Fig. 2:**
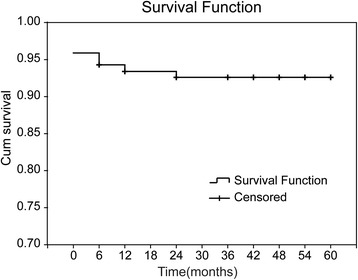
Five-year surival rates of TAPVC repair. At one month after TAPVC repair the surival rate was 95.1 %; there was none dead from two years to five years after operation. Actuarial 5-year survival is 92.6 %

The actuarial 5-year survival rates after TAPVC repair was 92.6 % (Fig. 2). Three patients died during follow-up owing to ventricular arrhythmia, right heart failure, and pneumonia at 5 months, 11 months, and 2 years after repair, respectively. A boy with myelodysplastic syndrome with a platelet count of 30,000/mL was treated with medicine. Seven patients were lost to follow-up. The others remained in New York Heart Association class I and II functional status, without medication. Echocardiographic studies revealed no gradient across the anastomosis in the patients.

**Fig. 3:**
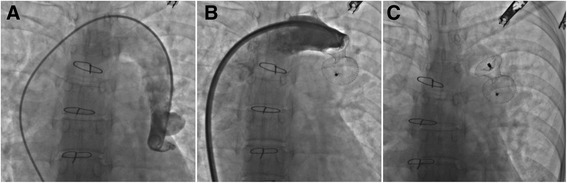
A case using an atrial septal defect amplatzer to close vertical vein six months after TAPVC repair. The patient was initially operated on with the vertical vein left open. Six months after the repair, the shunt was occluded with a atrial septal defect amplatzer by intervention. **a** Angiographic image of not ligated vertical vein; **b** Angiographic image when an ASD amplatzer implanted; **c**: Radiography after the amplatzer released

The patients with the vertical vein ligated at operation were all well except one boy whose pulmonary vascular resistance was 7.3 Wood units and who often had chest distress. When the LAP is higher than 15 mm Hg, loosening the snare of the vertical vein can reduce the LAP from 21 ± 5 mm Hg to 13 ± 3 mmHg (*P* < 0.05). In the patients with the vertical vein left intact, the shunt closed spontaneously in 8 and the shunt remained in 12. Among the 12 patients, 11 had a small left-to-right shunt and were monitored regularly. However, a 27-year-old female patient still had a large shunt, and the Qp/Qs value was 3.2 at 6 months after repair. We performed atrial septal defect closure by using of 34 mm (Shape Memory Co., Ltd, Shanghai; Fig. 3). The shunt and palpitation disappeared.

Supraventricular arrhythmia occurred in 19 patients who underwent supercardiac TAPVC repair through a biatrial incision, including atria extrasystole in 9 patients, relay of cardiac rhythm in 7, and atrial fibrillation in 3. The crista terminalis was incised, and the internodal tract was damaged during the repair through a biatrial incision. This may have contributed to the potential for arrhythmias. All of the patients recovered to normal sinus rhythm during 1 week after the operation. A 7-year-old girl died of ventricular arrhythmia 5 months after the operation. In another patient, atrial flutter persisted for 6 months after repair and subjected to electroversion to sinus rhythm by shock. None of the patients had arrhythmias.

## Discussion

Total anomalous pulmonary venous connection is a rare congenital malformation with an unfavorable natural prognosis. Only 20 % of patients survive the first year of life [13]. TAPVC in univentricular hearts included heterotaxy syndrome and pulmonary atresia, and such procedures as Fontan procedure, bidirectional Glenn procedure, systemic-pulmonary shunt, or pulmonary artery banding could be performed. TAPVC is usually associated with pulmonary venous obstruction and worse outcomes. Thus, we include a discussion on heterotaxy syndrome and pulmonary atresia.

For most patients with supracardiac TAPVC, we choose biatrial incision for total repair, which was described by Shumacker and King [14]. Another technique for dealing with the supracardiac type is a modified superior approach described by Serraf, especially for some patients younger than 1 year. This approach can be useful to enhance exposure during surgical repair and may contribute to an improved patient outcome. We used this approach successfully on patients with the infracardiac drainage type treated by making an biatrial incision. It can keep the heart in the normal position while keeping the anastomosis of the orifice unobstructed. While anastomosing the posterior orifice by lifting the heart upward and in the anterior direction, when the heart was returned, it was possible that anastomosis became kinked and turned, so the pulmonary vein drainage was blocked. It is interesting that only one patient had an infracardiac TAPVC, which may be due to the relatively older age of admission. This may be a reflection of socioeconomic status and late or no referral to the tertiary care center. This may explain why such a relatively old patient population would present with symptoms such as tachypnea, failure to thrive, and cyanosis.

Whether the vertical vein should be ligated at the time of total anomalous pulmonary venous repair is controversial. We considered that it may be well to leave the vertical vein open even though we obtained good results. One reason may be that the age in this group is somewhat old, and the pulmonary veins were not seriously obstructed. First, keeping the vertical vein open can alleviate the right ventricular load in the patient with serious pulmonary hypertension, and it can also alleviate the left ventricular load in the patient with poor ventricular compliance. Second, in infracardiac TAPVC, keeping the vertical vein open can also prevent hepatonecrosis. Last, the vertical vein can be closed by using the favored methods of adjustable means or intervention when the pulmonary pressure descends satisfactorily or the left ventricle is well trained. Chowdhury et al. preferred that an adjustable means can be used to close up the vertical vein after operation [15]. In our study, a woman aged 27 years was cured by intervention afterward. Kobayashi et al. also reported a similar case [16]. Although vertical vein ligation is recommended to close shunting, small left heart chambers may not always tolerate the immediate increase in blood flow [17]. Transcatheter vertical vein closure might render patients with high LAP suitable candidates for further hybrid approach.

In recent years, some centers reported that repair of TAPVC carried a relatively high early mortality (10–20 %) [2, 18]. However, in our department, only 6 deaths (4.9 %) occurred in the first month of repair. In the West, most patients who underwent TAPVC repair were neonates and young infants [11, 19, 20]. However, in our department, the age of most patients (85.2 %) who underwent TAPVC repair was older than 1 year. These patients rarely had obstructed drainage and moderate or severe pulmonary arterial hypertension. Thus, these patients (>1 year of age) seem naturally selected, representing 20 % of patients who survive beyond the first year of life. These were the important factors that might have contributed to such a low mortality rate in our department. The survival rates of patients with TAPVC and single-ventricle physiology were among the worst of all patients with congenital heart defects. Repeated operations for pulmonary venous obstruction were predominantly required in patients who underwent operation as neonates [5, 21]. We achieved good results with low mortality and few repeated operations, as the patients in this group were those with TAPVC and biventricular physiology. Those with pulmonary venous obstruction preoperation were indicated for emergency surgery, and we did not find post repair obstructions in pulmonary veins and an increase in early mortality rate.

Our data show that age younger than 1 year and weight less than 6 kg are risk factors, which may be due to emergency surgery. Eight patients underwent emergency surgery, of whom 7 were younger than 1 year and 6 weighed less than 6 kg. All of the patients who underwent emergency surgery had severe dyspnea and continuous metabolic acidosis preoperatively. When weaned from CPB, the patients usually had low blood pressure and became later prone to develop complications such as low cardiac output and infection with mechanical ventilator. Severe PAH often results in low cardiac output, arrhythmias, and lung complications. However, if the patients survived the perioperative period, they would have good results in the future.

## Conclusions

In conclusion, most patients who undergo repair with TAPVC can expect excellent outcomes with regard to survival, freedom from reintervention, and overall functional ability. However, the mortality rate is 22–25 % in the patients younger than 1 year of age or less than 6 kg in weight because of emergency surgery. If this pathology is not an emergency and the age and weight at presentation are older than 1 year and at least 6 kg, then one can obtain more-favorable results. However, the study is a retrospective review of medical records in a relatively small population. Its limitations also include the lack of a randomized controlled trial.
